# A transformer-based deep learning model for identifying the occurrence of acute hematogenous osteomyelitis and predicting blood culture results

**DOI:** 10.3389/fmicb.2024.1495709

**Published:** 2024-11-06

**Authors:** Yingtu Xia, Qiang Kang, Yi Gao, Jiuhui Su

**Affiliations:** ^1^Department of Orthopedics, The Second Hospital of Dalian Medical University, Dalian, Liaoning, China; ^2^Department of Radiology, Xing'an League People's Hospital of Inner Mongolia, Ulanhot, Inner Mongolia, China; ^3^Department of Laboratory Medicine, Shengjing Hospital of China Medical University, Shenyang, Liaoning, China; ^4^Department of Orthopaedics, Haicheng Bonesetting Hospital, Haicheng, Liaoning, China

**Keywords:** deep learning, osteomyelitis, blood culture, anti-infection treatment, pathogenic microorganism

## Abstract

**Background:**

Acute hematogenous osteomyelitis is the most common form of osteomyelitis in children. In recent years, the incidence of osteomyelitis has been steadily increasing. For pediatric patients, clearly describing their symptoms can be quite challenging, which often necessitates the use of complex diagnostic methods, such as radiology. For those who have been diagnosed, the ability to culture the pathogenic bacteria significantly affects their treatment plan.

**Method:**

A total of 634 patients under the age of 18 were included, and the correlation between laboratory indicators and osteomyelitis, as well as several diagnoses often confused with osteomyelitis, was analyzed. Based on this, a Transformer-based deep learning model was developed to identify osteomyelitis patients. Subsequently, the correlation between laboratory indicators and the length of hospital stay for osteomyelitis patients was examined. Finally, the correlation between the successful cultivation of pathogenic bacteria and laboratory indicators in osteomyelitis patients was analyzed, and a deep learning model was established for prediction.

**Result:**

The laboratory indicators of patients are correlated with the presence of acute hematogenous osteomyelitis, and the deep learning model developed based on this correlation can effectively identify patients with acute hematogenous osteomyelitis. The laboratory indicators of patients with acute hematogenous osteomyelitis can partially reflect their length of hospital stay. Although most laboratory indicators lack a direct correlation with the ability to culture pathogenic bacteria in patients with acute hematogenous osteomyelitis, our model can still predict whether the bacteria can be successfully cultured.

**Conclusion:**

Laboratory indicators, as easily accessible medical information, can identify osteomyelitis in pediatric patients. They can also predict whether pathogenic bacteria can be successfully cultured, regardless of whether the patient has received antibiotics beforehand. This not only simplifies the diagnostic process for pediatricians but also provides a basis for deciding whether to use empirical antibiotic therapy or discontinue treatment for blood cultures.

## 1 Introduction

Osteomyelitis is one of the common bone and muscle infections in children. Acute hematogenous osteomyelitis (AHO) is the most common form of this disease in pediatrics. In recent years, the incidence of osteomyelitis has been increasing annually (Walter et al., 2021). The incidence is generally higher in males compared to females, and lower limb infections are more prevalent than upper limb infections (Kremers et al., 2015; Disch et al., 2023). Typically, healthy bones have strong resistance to pathogen invasion. The occurrence of osteomyelitis mainly occurs through three mechanisms: direct inoculation, extension from adjacent lesions, and hematogenous dissemination. Additionally, in conditions of bone ischemia, trauma, or foreign bodies, pathogens are more likely to adhere to exposed bone locations, leading to bone infection. Most cases of osteomyelitis can be cured; however, a small number of affected children may experience discrepancies in limb length between the affected and unaffected sides (Liu et al., 2024).

The presentation of AHO varies, ranging from localized infections at a single epiphyseal site to multifocal infections accompanied by septic shock (Funk and Copley, 2017). Fever and pain are the most common manifestations of bone infections. Common signs of osteomyelitis include fever, pain, swelling, erythema, localized warmth, and varying degrees of functional impairment. The onset of symptoms can differ depending on the type of pathogen involved (Calvo et al., 2016). When the lower limb bones are affected, children often have difficulty bearing weight or may exhibit noticeable limping, whereas involvement of the pelvis may lead to a waddling gait. Overall, the functional impairment and the location of the infection are highly correlated (Dich et al., 1975). In fact, similar symptoms can be observed in various pediatric orthopedic conditions. For example, osteosarcoma (OSC) and Ewing’s sarcoma (EWS) are the most common primary malignant bone tumors in children and young adults, and they also present with significant pain and swelling (Wang et al., 2022). Fractures also present with localized pain, swelling, functional impairment, deformities, and abnormal movements. Considering that children may have difficulty responding accurately to physical examinations, confirming a diagnosis of osteomyelitis through simple procedures is more challenging.

Acute hematogenous osteomyelitis is often caused by pathogen infections, so identifying the type of pathogen early in the disease is crucial for treatment (Jahan et al., 2024). This not only directly guides the physician’s treatment but also provides psychological comfort to the family. Although *Staphylococcus aureus* is the most common pathogen in osteomyelitis (McNeil, 2020), pathogen culture is the gold standard for pathogen diagnosis, with blood culture being the most common method (Woods et al., 2021). To obtain accurate culture results, samples need to be taken before the use of antibiotics. However, in practice, it is difficult to ensure that patients have not self-medicated with antibiotics before admission. Bone biopsy or aspiration is also limited to the early stages of the disease. Thus, to achieve accurate culture results, discontinuing antibiotics is often necessary (Manz et al., 2018). For critically ill patients, discontinuing antibiotics to obtain accurate bacterial culture results is clearly impractical. Despite the stringent sampling requirements, not all children with acute osteomyelitis can successfully culture the pathogen (Section et al., 2015). Discontinuing antibiotics is often difficult for patients due to the potential risks of disease progression. Therefore, discontinuing medication to diagnose the pathogen type is a significant clinical challenge.

Artificial intelligence has been widely applied in the diagnosis and treatment of osteomyelitis. AI methods not only efficiently handle repetitive tasks and improve diagnostic efficiency but also explore complex relationships between medical information, mapping features to manifestations, and establishing quantitative relationships between medical information and clinical outcomes. A 2022 study classified acute osteomyelitis, chronic osteomyelitis, and Ewing’s sarcoma using patient X-ray images (Consalvo et al., 2022). Another 2022 study proposed a machine learning model based on clinical features and biomarkers to classify diabetic foot, necrotizing fasciitis, and osteomyelitis, trained and validated on a dataset of 1,581 samples (Kim et al., 2022). A 2024 study included 145 patients with diagnosed spinal infections undergoing metagenomic next-generation sequencing (mNGS) to differentiate pathogen types in iatrogenic vertebral osteomyelitis (IVO) and native vertebral osteomyelitis (NVO) (Gao et al., 2024). These studies demonstrate the potential of AI in the diagnosis and treatment of osteomyelitis.

This study explored the correlation between laboratory parameters and diseases commonly confused with acute hematogenous osteomyelitis. Based on this, we developed an intelligent diagnostic system based on clinical and laboratory features, which can classify patients into categories of acute hematogenous osteomyelitis, benign bone tumors, malignant bone tumors, and fractures. At the same time, we investigated the correlation between the ability to culture pathogenic bacteria and the number of hospital days, and established a deep learning model to predict whether pathogenic bacteria can be cultured. This model supports clinicians in deciding whether to discontinue antibiotics for blood culture purposes.

## 2 Materials and methods

### 2.1 Patient

**Figure 1:**
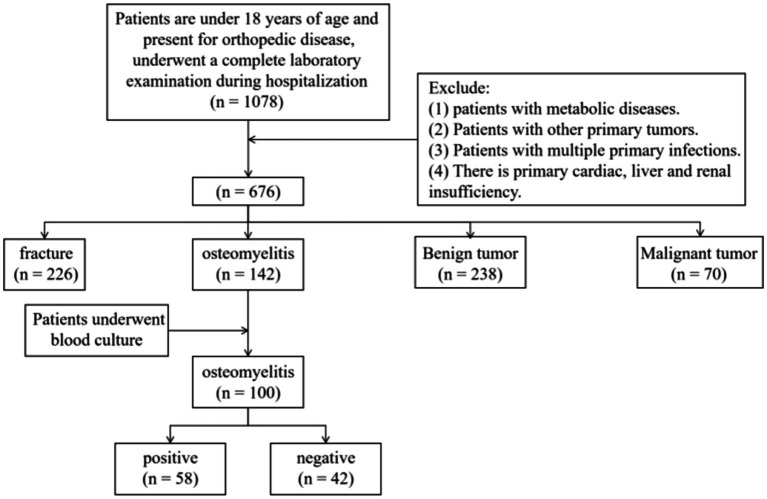
Inclusion and exclusion flowchart of patients in this study.

This study is a retrospective analysis that includes patients under 18 years of age who were hospitalized at our institution from January 1, 2016, to June 1, 2024. Baseline characteristics, including age and gender, were collected, as shown in Figure 1.

Inclusion criteria were as follows:

1. Age under 18 years at the time of admission.
2. Diagnosis of one of the following conditions: acute hematogenous osteomyelitis, benign bone tumor, malignant bone tumor, or fracture. AHO was diagnosed based on both laboratory indicators and clinical presentation. Benign and malignant bone tumors were diagnosed through pathology, while fractures were diagnosed through imaging or clear clinical manifestations (i.e., pain, deformity, and restricted movement).
3. For patients with AHO, at least one blood-based bacterial culture must have been performed during hospitalization.

Exclusion criteria were as follows:

1. Presence of metabolic diseases.
2. Presence of other primary tumors.
3. Presence of multiple primary infections.
4. Presence of primary heart, kidney, or liver dysfunction.

### 2.2 Laboratory parameters and blood culture

Collect laboratory data for the included population, The parameters related to blood cell counts include white blood cell count (WBC), red blood cell count (RBC), and platelets (PLT), all of which are obtained through the electrical impedance method (Coulter principle). Hemoglobin (HGB) is obtained using the sodium dodecyl sulfate hemoglobin colorimetric method. The white blood cell classification parameters include absolute eosinophil count (EO#, Eosinophil Count) and percentage (EO%, Eosinophil Percentage), absolute basophil count (BA#, Basophil Count) and percentage (BA%, Basophil Percentage), absolute lymphocyte count (LY#, Lymphocyte Count) and percentage (LY%, Lymphocyte Percentage), absolute monocyte count (MO#, Monocyte Count) and percentage (MO%, Monocyte Percentage), and absolute neutrophil count (NE#, Neutrophil Count) and percentage (NE%, Neutrophil Percentage). EO# and EO%, BA# and BA%, LY# and LY%, MO# and MO%, NE# and NE% are all obtained through VCS counting [V represents dual motor direct current characteristics (Coulter principle); C represents radio frequency conduction characteristics; S represents laser scattering]. Biochemical indicators include the albumin/globulin ratio (A/G), alanine aminotransferase (ALT), aspartate aminotransferase (AST), where AST is obtained through the reduced coenzyme method (NADH method). Albumin (AlbG) is obtained using the bromocresol green (BCG) method, and direct bilirubin (BilD) and total bilirubin (BilT) are obtained through the vanadate oxidation method. Gamma-glutamyl transferase (GGT) is obtained using the l-*γ*-glutamyl-3-carboxy-4-nitroaniline substrate method, and total protein (TP) is obtained using the biuret method. RH represents the Rh blood type (Rhesus Blood Type), obtained through the gel microcolumn method. Hematocrit (HCT), platelet count (PLT), mean platelet volume (MPV), plateletcrit (PCT), platelet distribution width (PDW), mean corpuscular volume (MCV), mean corpuscular hemoglobin (MCH), mean corpuscular hemoglobin concentration (MCHC), red cell distribution width (RDW), and unconjugated bilirubin (UNBIL) are all calculated values.

### 2.3 Blood culture

After the patient visits, blood samples should be collected as soon as possible. Draw 10 mL of blood from each arm of the patient for both aerobic and anaerobic blood cultures. Subsequently, send the blood culture bottles to the laboratory for testing. The detection involves monitoring whether bacteria consume nutrients in the culture bottles or produce new metabolites, triggering an alarm. Generally, if no alarm is triggered by the instrument within 5 days, the result is considered negative. If an alarm is triggered by the culture bottle, it is necessary to perform Gram staining microscopy and plate inoculation with the mixed solution from the culture bottle. For positive alarms in aerobic blood culture bottles, blood agar and MacConkey agar plates are commonly used. For positive alarms in anaerobic blood culture bottles, ensure anaerobic procedures and perform incubation in an anaerobic or microaerophilic environment.

### 2.4 Deep learning model

**Figure 2:**
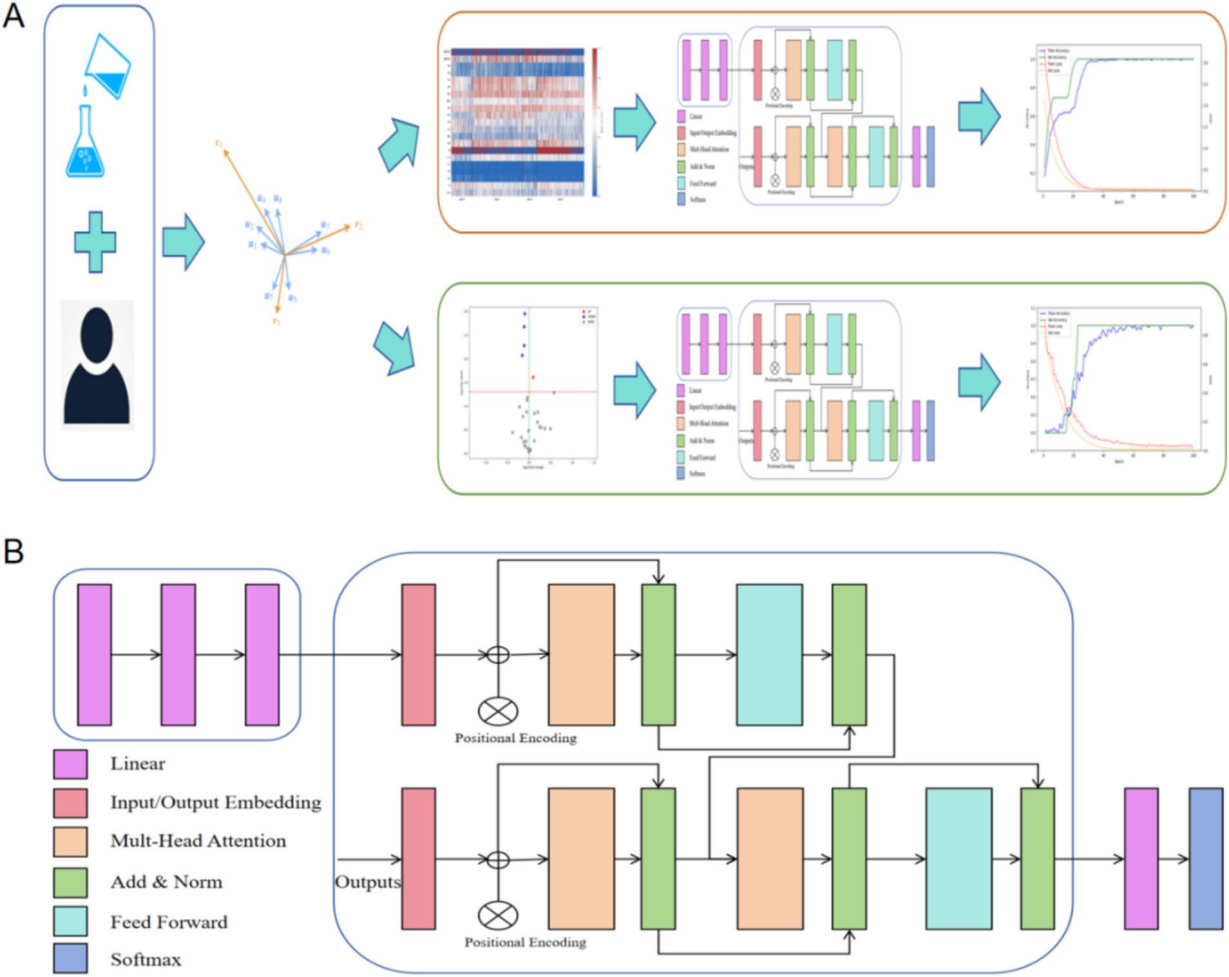
Research workflow of this study. (A) We constructed feature vectors using patients’ clinical characteristics and laboratory parameters, and utilized the deep learning architecture proposed in this study for classification tasks. (B) The structural diagram of the model in this study. Input was encoded through a standard ANN (with three linear layers and RELU activation function) and decoded through a transformer.

We developed a deep learning model structure to process laboratory parameters. The model consists of an ANN and a transformer. The transformer is a sequence-to-sequence model based on the attention mechanism, with the core idea of using self-attention to capture contextual relationships at different positions in the input sequence (Vaswani et al., 2017). We constructed feature vectors from clinical and laboratory data. The input is encoded through an ANN (including three linear layers with RELU activation functions) and then decoded by the transformer to classify the patients. For the two tasks in this study—differentiating AHO and identifying infection bacteria through blood cultures—we used the same model architecture, only changing the final fully connected layer to suit different tasks. The research workflow of this study is shown in Figure 2. The deep learning model in this study was trained on a workstation equipped with an NVIDIA RTX 4090 GPU (24GB VRAM) and 64GB of system memory. The training was based on PyTorch 2.4.1 and utilized CUDA 11.8 for acceleration.

### 2.5 Statistical analysis

Statistical analysis was performed using IBM SPSS software version 26.0. First, the Kolmogorov–Smirnov test was conducted to assess the normality of all data. For normally distributed data, the mean ± standard deviation was used for description, while for non-normally distributed data, the median (interquartile range) was used. Differences in continuous variables among multiple groups were analyzed using the Kruskal–Wallis *H* test, while categorical variables were analyzed using the chi-square test, with Cramér’s *V* used to quantify the strength of association. In the analysis between two groups, after testing for normality, the Mann–Whitney *U* test was applied to non-normally distributed data, and the *t*-test was used for normally distributed data. Differences in categorical variables were analyzed using the chi-square test, and Cramér’s *V* was used to quantify the association between categorical variables. Correlations between continuous variables were assessed using Pearson’s test for normally distributed data and Spearman’s test for non-normally distributed data. A *p*-value of <0.05 was considered statistically significant. Statistical plots were generated using Python’s matplotlib.

## 3 Results

### 3.1 Baseline

A total of 634 patients were included in this study, with variables having missing values greater than 20% being removed. Table 1 shows the clinical information and laboratory parameters of the included patients.

**Table 1:** Baseline information of patients.

	AHO/100	Benign bone tumor/238	Malignant bone tumor/70	Fracture/226
Sex	M67/F33	M146/F92	M31/F39	M153/F73
Gender	9.00 (5.00, 11.25)	11.50 (9.00, 14.00)	13.00 (10.25, 15.00)	9.00 (6.00, 11.00)
Length of hospital stay	19.50 (14.75, 27.00)	6.00 (5.00, 8.00)	15.00 (11.25, 19.00)	4.00 (3.00, 7.00)
EO#	0.15 (0.08, 0.22)	0.11 (0.10, 0.20)	0.10 (0.10, 0.20)	0.07 (0.02, 0.12)
EO%	2.40 (1.58, 3.73)	2.00 (1.30, 3.30)	1.70 (0.90, 2.90)	0.90 (0.20, 1.70)
HCT	33.95 (31.58, 36.95)	39.65 (37.60, 41.80)	34.45142	37.70 (35.25, 39.98)
HGB	112.00 (103.75, 121.00)	133.00 (124.00, 140.75)	115	126.00 (118.00, 133.00)
MCH	27.50 (26.48, 28.73)	28.40 (27.90, 29.50)	29.11285	28.40 (27.40, 29.00)
MCHC	331.18	335.00 (332.00, 340.00)	335.00 (329.00, 342.75)	335.00 (328.00, 339.00)
MCV	82.50 (80.00, 86.00)	85.00 (83.72, 87.83)	87.38571	85.00 (82.00, 87.00)
MPV	7.508	9.60 (8.50, 10.30)	9.26142	8.20 (7.50, 8.50)
PCT	0.2599	0.26394	0.278	0.25 (0.22, 0.27)
PLT	337.00 (285.75, 394.75)	280.50 (240.25, 323.75)	298.00 (260.25, 349.50)	298.00 (262.25, 342.75)
WBC	6.29 (4.98, 7.38)	6.89 (5.70, 8.00)	7.39 (5.93, 8.68)	8.41 (7.39, 11.82)
RBC	4.20 (3.80, 4.50)	4.40 (4.40, 4.71)	4.32 (4.00, 4.40)	4.40 (4.20, 4.80)
RH	1.00 (0.00, 1.00)	0.00 (0.00, 1.00)	0.00 (0.00, 0.00)	1.00 (1.00, 1.00)
A/G	1.30 (1.10, 1.60)	1.70 (1.60, 1.90)	1.586714286	1.68 (1.60, 1.90)
ALT	13.50 (10.00, 25.25)	13.00 (9.00, 18.00)	18.00 (12.00, 30.00)	13.00 (11.00, 15.00)
AST	21.00 (17.00, 25.00)	19.00 (15.00, 23.00)	19.00 (15.00, 26.00)	22.00 (21.00, 26.00)
GGT	17.00 (14.00, 28.00)	14.00 (11.00, 18.00)	21.00 (14.00, 30.25)	14.00 (12.00, 15.00)
TP	69.889	68.42521	64.57142	68.90 (68.22, 73.20)
Urea	4.31 (3.52, 4.77)	4.42 (3.81, 5.36)	4.00 (3.39, 4.59)	4.31 (3.83, 4.82)

### 3.2 Multicategory correlation analysis of laboratory parameters for AHO, benign bone tumors, malignant bone tumors, and fractures

**Figure 3:**
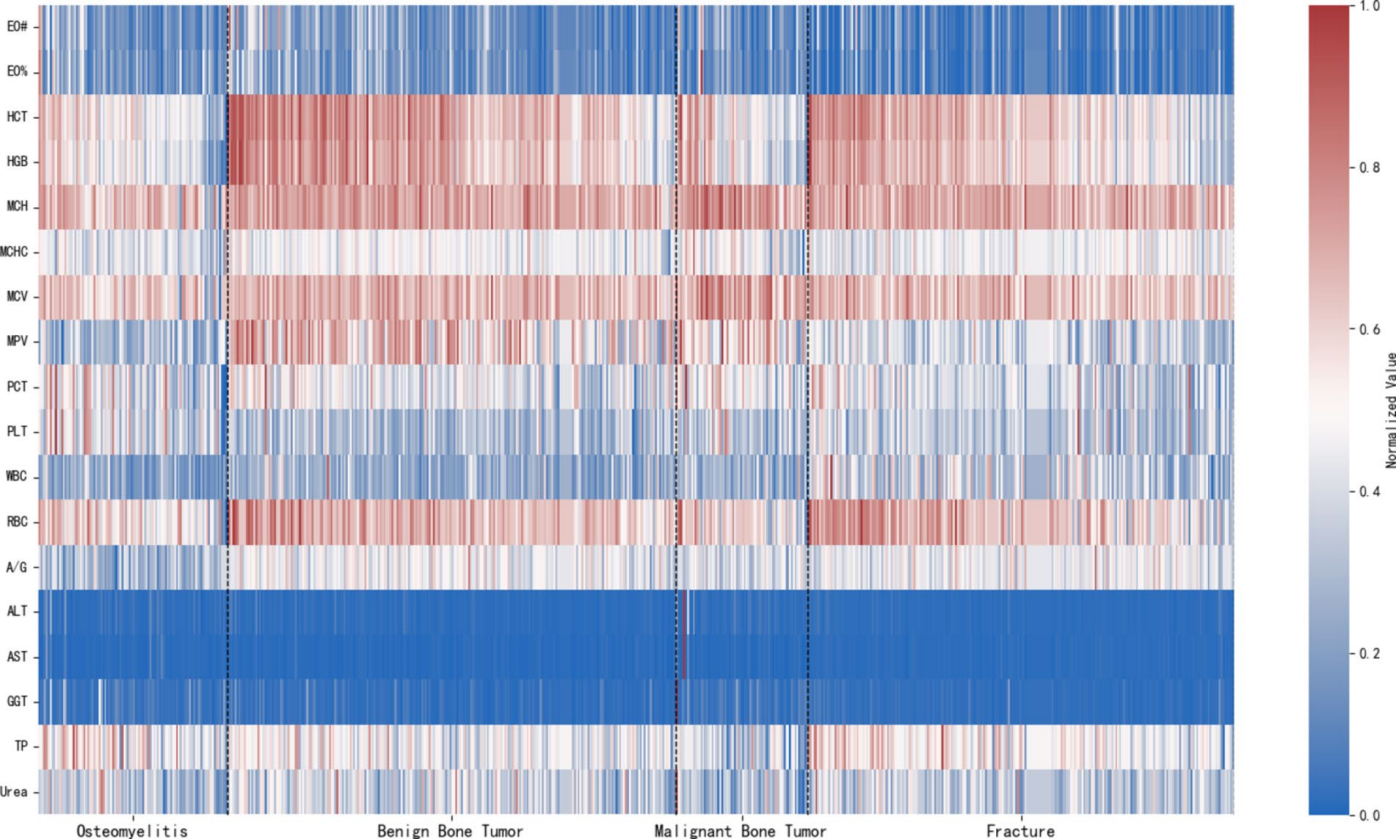
Expression of laboratory parameters in different disease types.

The expression of laboratory parameters in patients with different disease types is shown in Figure 3.

Correlation test results for disease types and clinical characteristics, as well as laboratory parameters, are presented in Tables 2, 3. We found that, among clinical characteristics, both gender and age were related to orthopedic disease types. In laboratory parameters, all parameters included in this study were related to orthopedic disease types.

**Table 2:** Association between categorical variables and disease classification using cramér’s *V*.

Indicator	Cramér’s *V*	*χ*^2^	*P*-value
Gender	0.14607	13.528	*0.00362
RH	0.41983	111.745	*0

*Has statistical significance.

**Table 3:** Analysis of variations in continuous variables across different disease groups.

Indicator	*H*-value	*P*-value	Indicator	*H*-value	*P*-value
Age	88.93294	*0	PCT	21.60439	*0
EO#	80.67734	*0	PLT	37.97623	*0
EO%	108.21281	*0	WBC	109.35881	*0
HCT	136.72223	*0	RBC	66.77762	*0
HGB	133.80915	*0	A/G	133.28753	*0
MCH	47.72300	*0	ALT	24.30355	*0
MCHC	14.29948	*0.00252	AST	43.32638	*0
MCV	49.52752	*0	GGT	68.77069	*0
MPV	229.19484	*0	TP	50.19360	*0
Urea	16.94605	*0.00072			

*Has statistical significance.

### 3.3 Multicategory deep learning of laboratory parameters for AHO, benign bone tumors, malignant bone tumors, and fractures

By removing columns with more than 20% missing values and imputing the missing values, a total of 21 common variables across the four diseases were used in modeling. The training, validation, and test sets were split in a 6:2:2 ratio, with baseline characteristics shown in Table 4.

**Table 4:** Baseline characteristics of the training, validation, and test sets for multicategory deep learning of laboratory parameters in AHO, benign bone tumors, malignant bone tumors, and fractures.

	Train/380				Validation/126				Test/128			
	AHO/70	Benign bone tumor/142	Malignant bone tumor/37	Fracture/131	AHO/18	Benign bone tumor/40	Malignant bone tumor/16	Fracture/52	AHO/12	Benign bone tumor/56	Malignant bone tumor/17	Fracture/43
Sex	M47/F23	M88/F54	M17/F20	M85/F46	M12/F6	M28/F12	M5/F11	M32/F20	M8/F4	M30/F26	M9/F8	M35/F8
Gender	9.00 (5.00, 11.00)	11.00 (8.00, 13.00)	12.64865	9.00 (6.00, 11.00)	8.50000	12.05000	14.00 (11.00, 14.25)	8.94118	7.66667	12.00 (9.50, 14.00)	11.82353	8.72093
EO#	0.15 (0.08, 0.22)	0.12 (0.10, 0.21)	0.10 (0.10, 0.20)	0.07 (0.02, 0.11)	0.11 (0.08, 0.14)	0.10 (0.10, 0.20)	0.10 (0.10, 0.23)	0.06 (0.01, 0.13)	0.22250	0.14 (0.10, 0.20)	0.10 (0.02, 0.19)	0.10 (0.03, 0.11)
EO%	2.60 (1.60, 3.80)	2.05 (1.20, 3.48)	2.00 (0.90, 3.20)	0.90 (0.20, 1.70)	2.05556	1.80 (1.50, 2.77)	2.00 (1.08, 3.10)	0.80 (0.15, 1.70)	3.65833	2.00 (1.60, 3.30)	1.41765	1.00 (0.30, 1.75)
HCT	34.40 (32.10, 37.20)	39.43873	35.23514	37.70 (35.45, 40.05)	34.06667	40.05 (37.70, 43.52)	32.57500	37.70 (34.80, 39.90)	31.28333	39.09455	31.70 (30.70, 35.80)	37.16977
HGB	113.00 (106.00, 122.00)	131.99296	117.94595	126.00 (119.00, 133.50)	113.44444	135.20000	109.62500	126.00 (115.50, 132.00)	103.75000	130.38182	113.64706	124.74419
MCH	27.30 (26.50, 28.60)	28.50 (27.90, 29.60)	29.07297	28.40 (27.40, 29.30)	27.80556	28.40 (27.93, 28.73)	28.98750	27.91961	26.74167	28.39818	29.31765	28.61163
MCHC	331.08696	335.00 (332.00, 339.75)	335.27027	335.00 (329.00, 339.00)	332.50000	336.75000	339.43750	333.58824	330.75000	334.92727	330.94118	335.69767
MCV	82.00 (80.00, 85.00)	85.51479	86.95946	85.00 (82.00, 87.00)	83.61111	85.00 (83.88, 86.27)	86.42500	83.70588	80.83333	85.18545	89.21765	85.30233
MPV	7.48116	9.60 (8.60, 10.30)	9.13514	8.20 (7.60, 8.50)	7.62222	9.54500	9.58750	8.01569	7.47500	9.31455	9.22941	8.50 (7.50, 8.60)
PCT	0.26536	0.26711	0.28 (0.25, 0.30)	0.25 (0.21, 0.27)	0.22778	0.25525	0.28125	0.26 (0.21, 0.28)	0.27750	0.26309	0.27294	0.26 (0.23, 0.28)
PLT	345.00 (292.00, 401.00)	279.00 (242.25, 331.25)	298.00 (267.00, 339.00)	298.00 (251.50, 337.50)	285.00 (254.25, 325.50)	274.00 (232.75, 298.00)	312.00000	316.54902	375.25000	296.00 (256.00, 324.50)	304.41176	303.00 (278.00, 342.00)
WBC	6.29 (5.04, 7.75)	6.90 (5.83, 8.54)	7.11541	8.40 (7.39, 11.75)	6.09000	6.48675	7.23875	9.97098	6.92917	7.03382	8.65471	8.27 (7.39, 9.96)
RBC	4.20 (3.90, 4.60)	4.42 (4.40, 4.71)	4.39 (4.00, 4.44)	4.40 (4.20, 4.80)	4.07778	4.40 (4.40, 4.65)	4.20 (4.04, 4.40)	4.40 (4.20, 4.80)	3.88333	4.48 (4.40, 4.80)	4.14294	4.40 (4.15, 4.75)
RH	1.00 (0.00, 1.00)	0.00 (0.00, 1.00)	0.00 (0.00, 0.00)	1.00 (1.00, 1.00)	0.00 (0.00, 1.00)	0.00 (0.00, 1.00)	0.00 (0.00, 0.00)	1.00 (1.00, 1.00)	0.50 (0.00, 1.00)	0.00 (0.00, 1.00)	0.00 (0.00, 0.00)	1.00 (0.00, 1.00)
A/G	1.30 (1.10, 1.60)	1.75690	1.57541	1.68 (1.60, 1.80)	1.45 (1.20, 1.66)	1.74300	1.57813	1.68 (1.50, 1.90)	1.38833	1.70 (1.60, 1.86)	1.61941	1.68 (1.60, 1.90)
ALT	14.00 (10.00, 25.00)	13.00 (9.00, 18.00)	21.00 (12.00, 30.00)	13.00 (11.50, 15.00)	19.50 (13.00, 37.00)	12.50 (9.75, 18.25)	18.00 (9.75, 34.50)	13.00 (10.00, 15.00)	13.25000	13.00 (9.00, 16.00)	14.00 (10.00, 24.00)	13.00 (11.50, 14.00)
AST	21.00 (17.00, 25.00)	20.00 (16.00, 24.00)	19.00 (17.00, 27.00)	22.00 (21.00, 26.00)	21.50 (19.25, 30.75)	19.00 (14.75, 22.25)	18.00 (14.75, 33.75)	21.00 (18.00, 26.50)	20.58333	19.00 (15.50, 21.00)	20.00 (14.00, 23.00)	22.00 (21.00, 26.00)
GGT	16.00 (14.00, 24.00)	13.00 (10.00, 18.00)	21.00 (14.00, 31.00)	14.00 (11.00, 15.00)	30.00 (15.00, 38.50)	14.00 (11.00, 20.25)	16.00 (12.75, 26.50)	14.00 (13.00, 17.00)	16.50 (13.25, 46.75)	14.00 (12.00, 18.00)	26.35294	13.00 (11.00, 14.00)
TP	69.50725	68.25634	64.69189	68.90 (67.60, 72.20)	70.67778	69.38500	65.40625	71.28824	70.50833	68.40000	63.52353	68.90 (67.55, 71.30)
Urea	4.24899	4.49 (3.88, 5.28)	3.61784	4.31 (3.79, 4.80)	4.21278	4.29 (3.76, 5.62)	4.06 (3.53, 4.68)	4.31 (4.01, 5.03)	4.90750	4.55418	4.41529	4.22070

**Figure 4:**
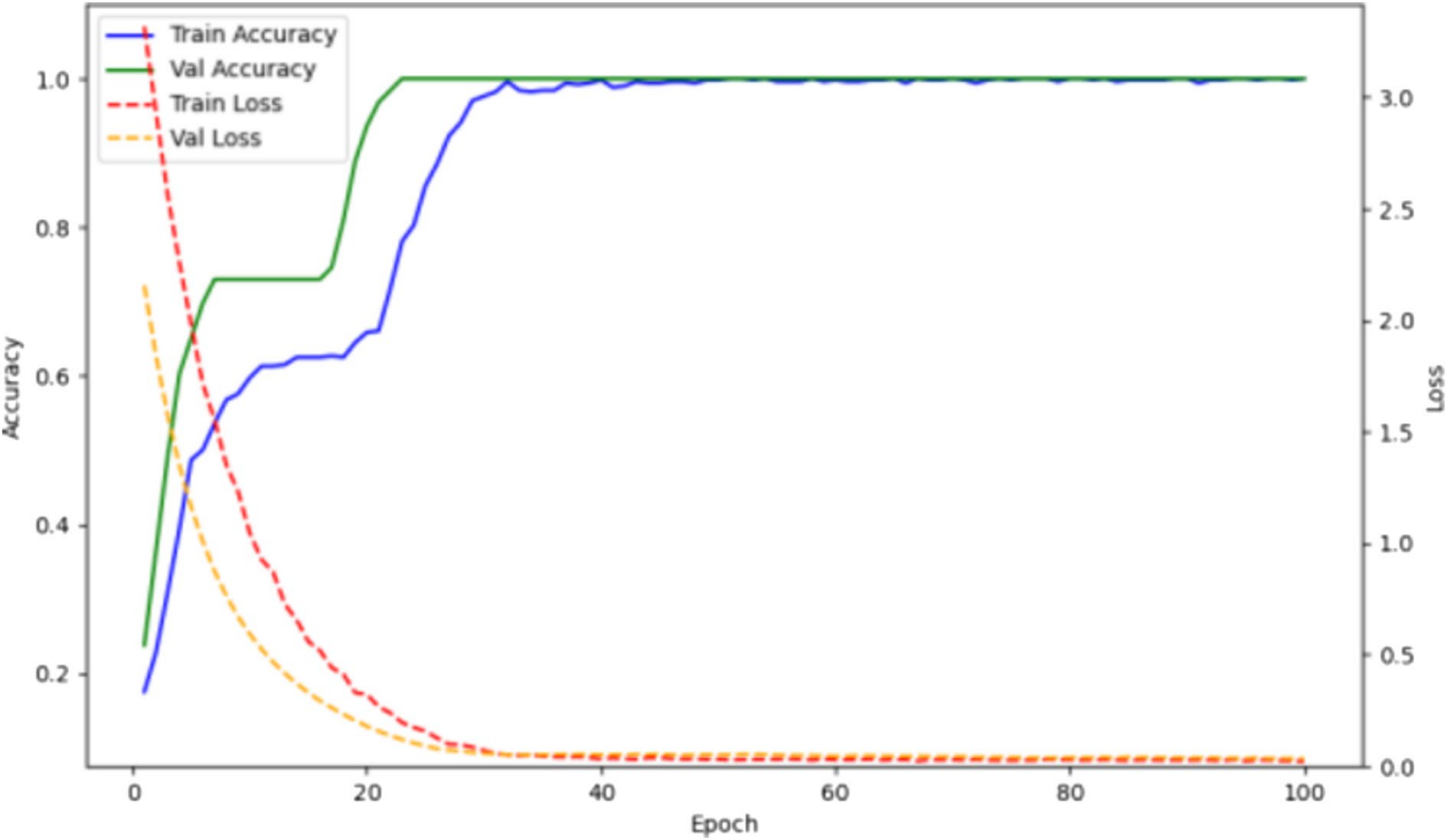
Accuracy and loss curves of the multicategory deep learning model for laboratory parameters in AHO, benign bone tumors, malignant bone tumors, and fractures on the training and validation sets.

The training results are shown in Figure 4. Over 32 epochs, the model achieved optimal performance on the validation set and attained an accuracy of 1.0 on the test set.

### 3.4 Correlation between hospitalization duration for AHO and laboratory parameters

As shown in Tables 5, 6, although AHO is more common in males, there is no correlation between the patient’s gender or age and their hospitalization duration. Among the laboratory parameters, RDW is positively correlated with the length of hospital stay, while HCT, HGB, MCH, MCV, and RBC are negatively correlated with the length of hospital stay, with a *p*-value of <0.05. This indicates a strong link between the patient’s hospitalization duration and their red blood cell physiological state. Although inflammation markers can assess disease severity, they are not directly related to the patient’s hospitalization duration. Liver function also shows no correlation with the hospitalization duration for AHO. The correlation between bacterial culture results and clinical information or laboratory parameters in AHO patients reveals statistical differences in HCT, HGB, RDW, A/G, and AlbG. Additionally, there is a significant difference in the length of hospital stay between the negative and positive culture sample groups. As shown in Table 7, patients with positive cultures have a longer hospital stay.

**Table 5:** Correlation between continuous variables and hospitalization duration.

Indicator	Correlation	*P*-value	Indicator	Correlation	*P*-value
Age	0.00963	0.92427	LY#	−0.02446	0.80911
EO#	0.04269	0.67326	LY%	0.05748	0.56999
EO%	0.09702	0.33692	MO#	0.08611	0.39429
HCT	−0.33353	*0.00070	MO%	0.07880	0.43583
HGB	−0.35001	*0.00036	NE#	−0.07093	0.48315
MCH	−0.25294	*0.01112	NE%	−0.09425	0.35096
MCHC	−0.15533	0.12281	RDW	0.37916	*0.00010
MCV	−0.26286	*0.00824	A/G	−0.03404	0.73673
MPV	0.03388	0.73787	ALT	0.02556	0.80071
PCT	−0.05340	0.59771	AST	−0.15158	0.13222
PLT	0.08313	0.41091	AlbG	−0.06810	0.50082
WBC	−0.05722	0.57176	BilD	0.01904	0.85087
RBC	−0.22104	*0.02710	BilT	−0.05883	0.56096
PDW	0.04390	0.66454	GGT	−0.07641	0.44987
BA#	0.08405	0.40572	TP	−0.08939	0.37648
BA%	0.06003	0.55300	UNBIL	−0.12966	0.19854

*Has statistical significance.

**Table 6:** Analysis of variations in hospitalization duration across different categorical variables.

Indicator	*U*-value	*P*-value
Bacterial culture	1636.0	*0.00362
Gender	1272.0	0.22296
RH	943.0	0.07069

*Has statistical significance.

**Table 7:** Baseline information for AHO bacterial culture.

	Negative/42	Positive/58
Gender	M26/F16	M41/F17
Age	8.00 (4.00, 11.00)	10.00 (6.00, 12.00)
Length of hospital stay	16.00 (12.00, 23.00)	21.00 (16.00, 28.00)
EO#	0.14 (0.08, 0.27)	0.15 (0.09, 0.21)
EO%	2.40 (1.55, 4.00)	2.45 (1.60, 3.55)
HCT	35.25476	32.65172
HGB	119.00 (110.00, 125.00)	107.91379
MCH	27.65714	27.50 (26.10, 28.75)
MCHC	332.40476	330.29310
MCV	83.21429	82.00 (78.50, 85.00)
MPV	7.52857	7.49310
PCT	0.25905	0.26052
PLT	333.50 (288.25, 376.00)	352.81034
WBC	5.99 (4.90, 7.69)	6.36 (5.03, 7.30)
RBC	4.21429	4.10 (3.80, 4.45)
PDW	16.20952	16.20 (15.93, 16.40)
BA#	0.03310	0.03 (0.02, 0.04)
BA%	0.60 (0.40, 0.70)	0.50 (0.40, 0.70)
LY#	2.35 (1.80, 3.05)	2.20 (1.80, 2.90)
LY%	41.83571	37.92931
MO#	0.40 (0.33, 0.60)	0.40 (0.40, 0.60)
MO%	7.60000	7.35 (5.62, 8.10)
NE#	2.70 (2.02, 3.75)	3.10 (2.02, 4.20)
NE%	46.96429	51.12759
RDW	14.44524	14.60 (14.03, 16.10)
RH	1.00 (0.00, 1.00)	1.00 (0.00, 1.00)
A/G	1.30 (1.20, 1.40)	1.22759
ALT	16.00 (10.00, 20.00)	16.00 (11.25, 30.75)
AST	20.00 (18.25, 25.75)	20.00 (16.00, 23.00)
AlbG	39.55 (38.50, 41.30)	38.40 (36.05, 39.27)
BilD	1.60 (1.30, 2.27)	1.70 (1.30, 2.85)
BilT	4.75 (3.17, 6.35)	4.80 (3.82, 6.80)
GGT	18.00 (13.25, 23.00)	19.50 (16.25, 30.00)
TP	70.06905	69.91552
UNBIL	3.00 (2.02, 4.28)	3.00 (2.42, 4.07)

### 3.5 Correlation analysis of bacterial culture results in AHO

Baseline information for AHO patients is shown in Table 7. Additionally, among the patients with positive cultures, the identified strains included 51 *Staphylococcus aureus*, 1 *Acinetobacter baumannii*, 1 *Staphylococcus epidermidis*, 1 *Bacillus subtilis*, 1 *Achromobacter xylosoxidans*, 1 Klebsiella, 1 Malassezia furfur, and 1 *Pseudomonas aeruginosa*.

**Figure 5:**
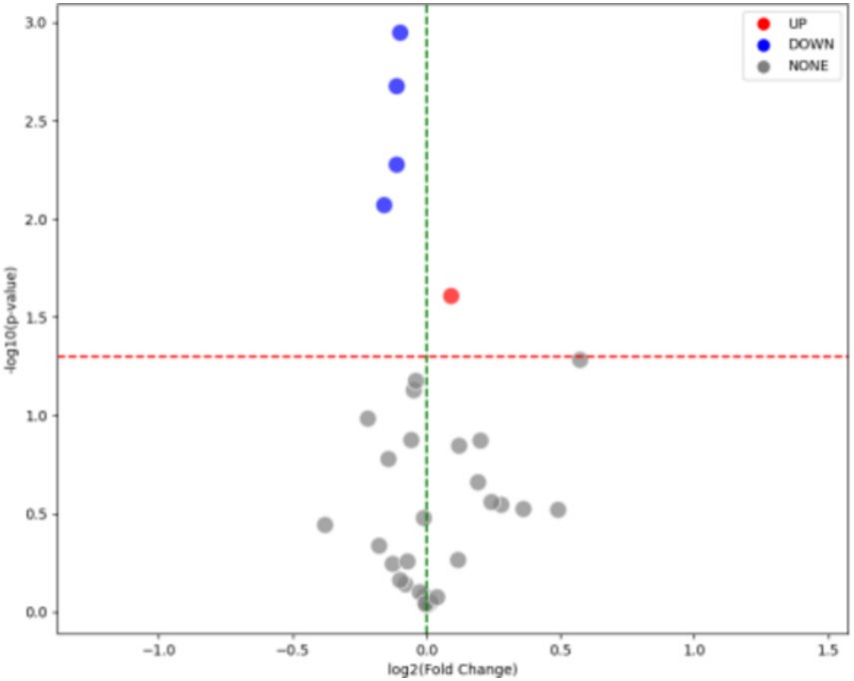
Upregulation and downregulation of parameters in the groups with or without detection of pathogenic bacteria in AHO patients: parameters were adjusted as follows: RDW was upregulated, while A/G, HGB, HCT, and AlbG were downregulated.

As shown in Tables 8, 9, there are no significant differences in gender and RH between the negative and positive culture sample groups. Only HGB, A/G, AlbG, GGT, and HCT showed significant differences between the negative and positive culture sample groups. According to Table 7, the positive culture group had lower levels of HGB, A/G, AlbG, and HCT, and higher levels of GGT. The upregulation and downregulation situations in the two groups are illustrated in Figure 5.

**Table 8:** Association between categorical variables and bacterial culture negative/positive groups using cramér’s *V*.

Indicator	Cramér’s *V*	*χ*^2^	*P*-value
Gender	0.07067	0.49937	0.47978
RH	0.05376	0.28907	0.59082

**Table 9:** Analysis of variations in continuous variables between bacterial culture negative and positive groups.

Indicator	*U*-statistic	*P*-value
Age	1,416	0.16587
EO#	1,187	0.83119
EO%	1137.5	0.57622
HGB	751	*0.00111
MCH	1,073	0.31273
MCV	1058.5	0.26504
PLT	1,257	0.78801
WBC	1238.5	0.88891
RBC	982.5	0.10002
PDW	1199.5	0.89968
BA#	1,251	0.81698
BA%	1165.5	0.71384
LY%	1,012	0.15122
MO#	1193.5	0.86423
MO%	1,124	0.51365
NE%	1433.5	0.13321
RDW	1,431	0.13761
A/G	896.5	*0.02347
ALT	1381.5	0.25372
AST	1,071	0.30436
AlbG	762.5	*0.00146
BilD	1444.5	0.11271
BilT	1,362	0.31502
GGT	1516.5	*0.03702
UNBIL	1284.5	0.64422

Indicator	*t*-statistic	*P*-value
HCT	3.15776	*0.00211
MCHC	0.97213	0.33338
MPV	0.21465	0.83049
PCT	−0.10334	0.91791
LY%	1.39233	0.16697
NE%	−1.47695	0.14289
TP	0.11779	0.90647

*Has statistical significance.

### 3.6 Deep learning model for detecting pathogenic bacteria in AHO

By removing columns with more than 20% missing values and filling in the missing values, 34 common variables from both bacterial culture negative and positive AHO patients were used for modeling. The baseline features are shown in Table 10.

**Table 10:** Baseline features of the deep learning model for pathogen detection in training, validation, and test sets.

	Train		Validation		Test	
	Negative	Positive	Negative	Positive	Negative	Positive
Sex	M15/F13	M 25/F7	M 3/F3	M 8/F6	M 8/F1	M 8/F3
Gender	6.51852	10.00 (6.75, 11.25)	11.40000	7.85714	9.75000	11.00 (8.50, 13.00)
EO#	0.15 (0.08, 0.27)	0.14 (0.09, 0.20)	0.07200	0.13 (0.09, 0.17)	0.26375	0.18636
EO%	2.60 (1.15, 4.00)	2.55 (1.68, 3.08)	2.10 (1.10, 2.10)	1.60 (1.10, 4.28)	4.12500	3.23636
HCT	35.19259	32.66875	34.18000	34.32857	36.16250	29.70909
HGB	116.88889	108.46875	106.80000	112.92857	122.12500	104.00 (85.50, 108.50)
MCH	27.67778	27.65 (26.60, 29.02)	27.14000	27.28571	28.12500	24.42727
MCHC	332.33333	331.90625	328.20000	328.64286	337.62500	327.45455
MCV	83.33333	82.00 (80.75, 85.25)	82.60000	83.00000	83.37500	74.63636
MPV	7.57407	7.39688	7.62000	7.34286	7.33750	7.85455
PCT	0.24 (0.23, 0.29)	0.26094	0.22600	0.24929	0.27375	0.27727
PLT	331.00 (289.50, 365.50)	336.00 (289.75, 407.75)	301.40000	342.35714	379.37500	359.90909
WBC	6.78630	6.35 (4.83, 6.96)	4.78000	7.03786	6.29625	6.27636
RBC	4.22593	3.99688	3.94000	4.13571	4.35000	4.04545
PDW	16.26667	16.24063	16.10000	15.98571	16.07500	16.34545
BA#	0.03556	0.03 (0.02, 0.04)	0.02800	0.04071	0.03 (0.02, 0.03)	0.03091
BA%	0.60 (0.40, 0.70)	0.50 (0.40, 0.72)	0.64000	0.59286	0.55000	0.49091
LY#	2.70 (1.90, 3.30)	2.24375	1.56000	2.56429	2.11250	2.30 (1.80, 2.60)
LY%	45.11481	37.85000	32.88000	38.67857	34.76250	39.34545
MO#	0.40 (0.35, 0.60)	0.48125	0.46000	0.40 (0.40, 0.47)	0.45000	0.43636
MO%	7.31481	7.75 (6.57, 8.30)	9.00000	6.65 (5.38, 7.95)	7.12500	6.87273
NE#	2.70 (2.00, 3.40)	3.05 (2.18, 3.88)	2.86000	3.76429	3.43750	3.18182
NE%	43.90741	51.00313	56.04000	50.32857	53.46250	50.05455
RDW	14.57037	14.60 (14.10, 15.72)	15.28000	14.45000	13.65000	17.81818
RH	1.00 (0.00, 1.00)	0.50 (0.00, 1.00)	1.00 (1.00, 1.00)	1.00 (0.00, 1.00)	1.00 (0.75, 1.00)	1.00 (0.50, 1.00)
A/G	1.30 (1.20, 1.45)	1.18125	1.48000	1.31429	1.27500	1.22727
ALT	15.00 (9.00, 20.00)	16.00 (11.00, 28.25)	23.80000	17.00 (12.25, 40.25)	14.50000	16.00 (13.50, 23.50)
AST	23.00 (19.50, 26.00)	19.00 (16.00, 22.25)	19.40000	20.50 (19.00, 26.75)	18.12500	20.00 (17.00, 21.50)
AlbG	39.30 (38.45, 41.20)	36.88438	41.20000	37.76429	39.95000	38.60 (37.35, 40.55)
BilD	1.70 (1.30, 2.45)	1.70 (1.30, 2.70)	1.40 (1.30, 2.30)	1.60 (1.30, 2.55)	1.40000	1.70 (1.65, 2.40)
BilT	5.50000	4.80 (3.10, 6.42)	3.70 (3.40, 5.40)	5.19286	3.98750	4.80 (4.60, 6.95)
GGT	15.00 (12.50, 20.00)	21.00 (16.75, 30.00)	26.40000	29.00 (17.50, 50.25)	21.00000	19.18182
TP	69.84444	70.17500	69.44000	67.52143	71.50000	71.26364
UNBIL	3.52222	3.00 (1.95, 4.17)	2.60 (2.30, 3.10)	3.00 (2.60, 3.33)	2.57500	3.20 (2.75, 4.05)

**Figure 6:**
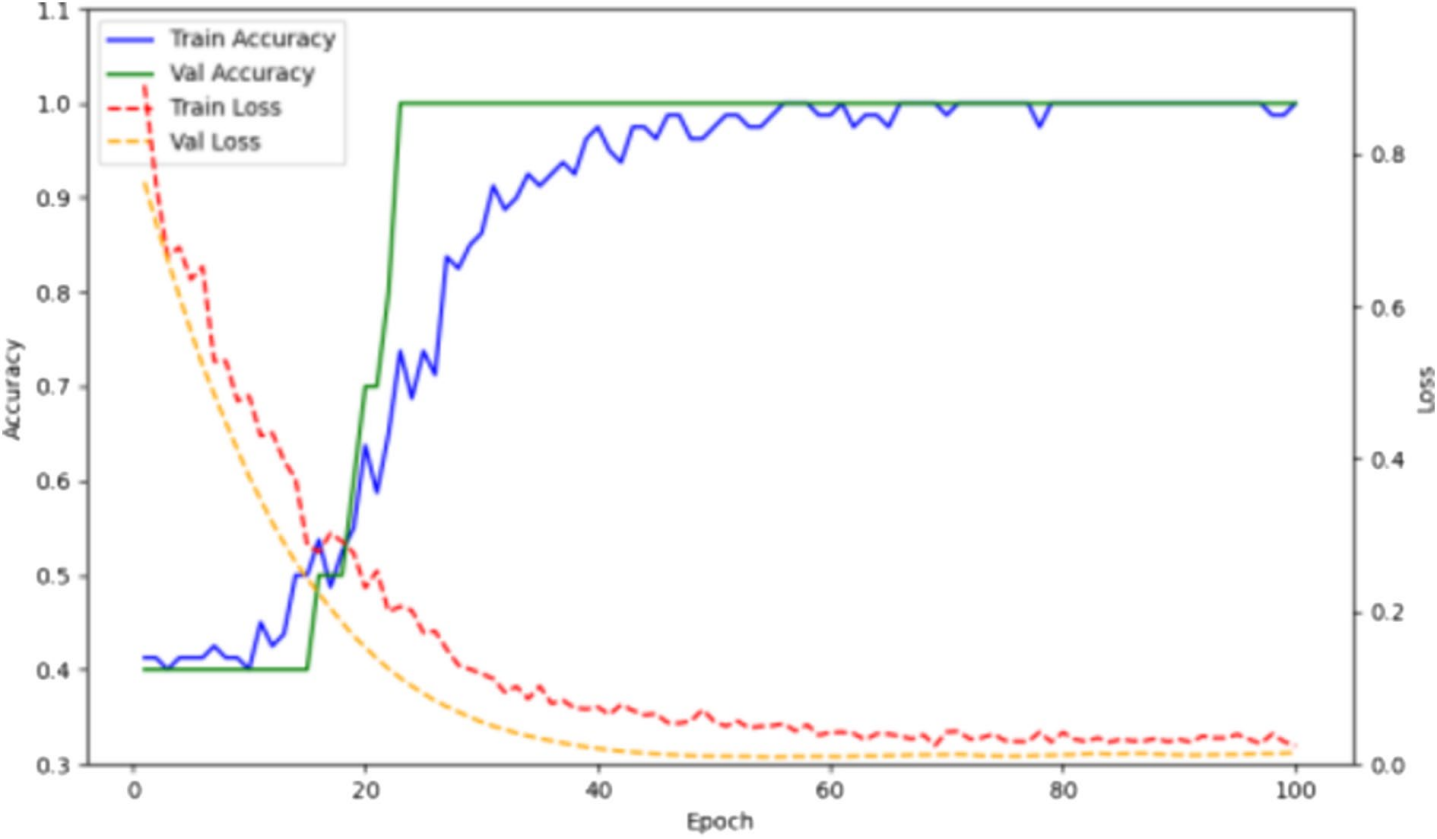
Accuracy and loss curves of the deep learning model for pathogen detection on training and validation sets.

The training/testing ratio was 6:2:2. The training results are shown in Figure 6. Over 58 epochs, the model achieved optimal performance on the validation set and an accuracy of 1.0 on the test set.

## 4 Discussion

Acute hematogenous osteomyelitis is a challenging diagnosis in pediatric emergency departments. The condition can develop gradually over a few days but typically manifests within 2 weeks. Patients may present with localized symptoms such as redness, swelling, and fever at the infection site. They might experience dull pain, with or without movement, and sometimes systemic symptoms such as fever or chills. In subacute cases, some patients may exhibit generalized discomfort, mild pain over several weeks accompanied by slight fever or other systemic symptoms (Schmitt, 2017). The variety of symptoms and the difficulty children have in clearly describing their condition make the diagnosis and treatment of osteomyelitis quite challenging (Stephan et al., 2022). In this study, we propose a diagnostic model consisting of two deep learning models that can accurately diagnose AHO in children and predict whether the pathogenic bacteria can be identified through blood cultures. This study not only provides clinicians with a straightforward method for confirming AHO but also offers support for decisions regarding the necessity of stopping antibiotics for bacterial culture.

There are several methods for diagnosing AHO, with blood cultures and X-rays currently being strongly recommended. However, both methods have their drawbacks. Blood cultures require a lengthy period to yield results and cannot always ensure accuracy (Doern et al., 2019). X-rays, while simple, quick, and safe, have low sensitivity (Zaki and Morrison, 2024). Additionally, MRI often requires sedation for pediatric patients, and collecting samples from the affected area for bacterial culture through invasive methods faces the same issues as blood culture (Dartnell et al., 2012; Dong et al., 2019). In addition to these two types of examination methods, recent research by Paliwal et al. (2021) has revealed the diagnostic capability of ultrasound for acute hematogenous osteomyelitis (AHO). By assessing the accumulation of deep soft tissue fluid around the bones in AHO cases, rapid diagnosis can be achieved. This method is also expected to advance the diagnosis of AHO (Paliwal et al., 2021).

Additionally, Stephan et al. (2022) identified elevated C-reactive protein (CRP) and erythrocyte sedimentation rate (ESR) as the most sensitive laboratory markers in pediatric emergency settings. In the study by Manz et al. (2020), elevated CRP was also found to be associated with poor long-term outcomes in AHO. Based on previous studies, Stephan et al. (2024) also demonstrated in subsequent experiments that elevated CRP and ESR are closely related to poor long-term prognosis in AHO. In this study, we propose a method for diagnosing AHO through laboratory parameters. By employing a multiclass model structure, it can effectively diagnose several common orthopedic conditions, significantly enhancing diagnostic efficiency. Considering that the model in this study needs to differentiate between osteomyelitis and other orthopedic diseases, and that C-reactive protein and erythrocyte sedimentation rate are not essential laboratory parameters for orthopedic diseases, they were not included in this study.

Different types of pathogenic bacteria may lead to variations in hospital length of stay. A 2023 study indicated that there is a difference in hospital stay duration for osteomyelitis caused by methicillin-resistant *Staphylococcus aureus* (MRSA) and methicillin-sensitive *Staphylococcus aureus* (MSSA) (Wen et al., 2023). Additionally, children with acute osteomyelitis who are Black, Hispanic, or of other races and ethnicities have longer hospital stays compared to White children (Campbell et al., 2023). In our study, we found that the infection level and liver function levels of patients were not associated with the length of hospital stay. Considering that in China, the length of hospital stay is highly correlated with the patient’s recovery of physical health and is less influenced by the wishes of the patients and their families, we believe that the status of the red blood cells is an important factor affecting the recovery process of AHO. This study found a positive correlation between RDW and length of hospital stay, while HCT, HGB, MCH, MCV, and RBC showed a negative correlation with length of hospital stay. This may be due to the important role of red blood cells in oxygen transport and bone tissue repair. If there is an insufficient number of red blood cells (as seen in anemia) or abnormal red blood cell function, the bone tissue may not receive enough oxygen, which can delay repair.

The Pediatric Infectious Diseases Society and the Infectious Diseases Society of America recommend performing blood cultures before the use of antibiotics (Woods et al., 2021). Despite the presence of clear symptoms, the detection rate of blood cultures remains unsatisfactory, and this rate rarely exceeds 60% in various cohorts (Russell et al., 2015; McNeil et al., 2019). Although sampling from adjacent infected sites can somewhat improve the detection rate, it does not provide an overwhelming advantage over blood cultures (Athey et al., 2019). In practical medical work, patients often have already been on oral antibiotics for some time by the time they arrive at the hospital. Once effective antibiotics are started, the detection rate of blood cultures usually drops rapidly within a few hours of exposure. Therefore, deciding whether to stop antibiotics for culture testing is a challenging decision for physicians. Previous studies have analyzed different clinical features as independent predictors of positive blood cultures (Burns et al., 2023). In this study, our model accurately predicts whether pathogens can be identified through blood cultures. This provides guidance for clinicians on whether to proceed with empirical antibiotic treatment. This study reveals that, in addition to affecting the recovery process of AHO patients, red blood cell status also influences the results of bacterial culture. This may be related to how red blood cell status affects the overall function of the immune system, thereby impacting the proliferation and distribution of bacteria within the body. Abnormal red blood cell function may weaken the immune response to infections, allowing pathogens to become localized in tissues and making them more difficult to detect in blood cultures.

Despite this, the study has some limitations. First, it is a single-center study, including both laboratory parameters and blood culture results. This suggests that the study may have potential issues with generalizability. Second, the study only established a classification method between acute hematogenous osteomyelitis and benign bone tumors, malignant bone tumors, and fractures, without exploring classifications for different subtypes and disease progressions of osteomyelitis, which is also due to a lack of data. Third, while the study predicted whether pathogens could be successfully cultured from patients, it did not further classify the cultured pathogens. Fourth, this study only included laboratory parameters from AHO patients, while the diagnosis of AHO can also rely on imaging information. Due to a lack of data, this study did not include any medical imaging information. Therefore, the next step for this research is to collect data from multiple centers, expand the cohort, and include more data modalities.

## 5 Conclusion

Early laboratory parameters can accurately diagnose whether pediatric patients have acute hematogenous osteomyelitis. Laboratory parameters can describe the severity of acute hematogenous osteomyelitis and are somewhat correlated with the patient’s length of hospital stay. Early laboratory parameters can predict whether a patient’s blood sample will successfully culture pathogens, thereby guiding clinical decision-making, indirectly improving clinical outcomes, and shortening the hospital stay.

## Data Availability

The raw data supporting the conclusions of this article will be made available by the authors, without undue reservation.

## References

[ref1] Athey A. G. Mignemi M. E. Gheen W. T. Lindsay E. A. Jo C. H. Copley L. A. (2019). Validation and modification of a severity of illness score for children with acute Hematogenous osteomyelitis. J. Pediatr. Orthop. 39, 90–97. doi: 10.1097/bpo.0000000000000879, PMID: 27741035

[ref2] Burns J. D. Upasani V. V. Bastrom T. P. Baldwin K. D. Schoenecker J. G. Shore B. J.. (2023). Age and CRP associated with improved tissue pathogen identification in children with blood culture negative osteomyelitis: results from the CORTICES multicenter database. J. Pediatr. Orthop. 43, e603–e607. doi: 10.1097/bpo.0000000000002448, PMID: 37278086

[ref3] Calvo C. Núñez E. Camacho M. Clemente D. Fernández-Cooke E. Alcobendas R.. (2016). Epidemiology and management of acute, uncomplicated septic arthritis and osteomyelitis: Spanish multicenter study. Pediatr. Infect. Dis. J. 35, 1288–1293. doi: 10.1097/inf.0000000000001309, PMID: 27455444

[ref4] Campbell J. I. Shanahan K. H. Bartick M. Ali M. Goldmann D. Shaikh N.. (2023). Racial and ethnic differences in length of stay for US children hospitalized for acute osteomyelitis. J. Pediatr. 259:113424. doi: 10.1016/j.jpeds.2023.113424, PMID: 37084849 PMC10527861

[ref5] Consalvo S. Hinterwimmer F. Neumann J. Steinborn M. Salzmann M. Seidl F.. (2022). Two-phase deep learning algorithm for detection and differentiation of Ewing sarcoma and acute osteomyelitis in paediatric radiographs. Anticancer Res. 42, 4371–4380. doi: 10.21873/anticanres.15937, PMID: 36039445

[ref6] Dartnell J. Ramachandran M. Katchburian M. (2012). Haematogenous acute and subacute paediatric osteomyelitis: a systematic review of the literature. J. Bone Joint Surg. Br. 94, 584–595. doi: 10.1302/0301-620x.94b5.28523, PMID: 22529075

[ref7] Dich V. Q. Nelson J. D. Haltalin K. C. (1975). Osteomyelitis in infants and children. A review of 163 cases. Am. J. Dis. Child. 129, 1273–1278. doi: 10.1001/archpedi.1975.02120480007004 1190158

[ref8] Disch K. Hill D. A. Snow H. Dehority W. (2023). Clinical outcomes of pediatric osteomyelitis. BMC Pediatr. 23:54. doi: 10.1186/s12887-023-03863-z, PMID: 36732705 PMC9896664

[ref9] Doern G. V. Carroll K. C. Diekema D. J. Garey K. W. Rupp M. E. Weinstein M. P.. (2019). Practical guidance for clinical microbiology laboratories: a comprehensive update on the problem of blood culture contamination and a discussion of methods for addressing the problem. Clin. Microbiol. Rev. 33:e00009-19. doi: 10.1128/cmr.00009-19, PMID: 31666280 PMC6822992

[ref10] Dong S. Z. Zhu M. Bulas D. (2019). Techniques for minimizing sedation in pediatric MRI. J. Magn. Reson. Imaging 50, 1047–1054. doi: 10.1002/jmri.26703 30869831

[ref11] Funk S. S. Copley L. A. (2017). Acute Hematogenous osteomyelitis in children: pathogenesis, diagnosis, and treatment. Orthop. Clin. North Am. 48, 199–208. doi: 10.1016/j.ocl.2016.12.007 28336042

[ref12] Gao Q. Liu Q. Zhang G. Lu Y. Li Y. Tang M.. (2024). Identification of pathogen composition in a Chinese population with iatrogenic and native vertebral osteomyelitis by using mNGS. Ann. Med. 56:2337738. doi: 10.1080/07853890.2024.2337738, PMID: 38590185 PMC11005868

[ref13] Jahan T. A. Lapin N. A. O'Connell M. T. Jo C. Ma Y. Tareen N. G.. (2024). Accelerated severity of illness score enhances prediction of complicated acute hematogenous osteomyelitis in children. Pediatr. Infect. Dis. J. doi: 10.1097/inf.0000000000004535, PMID: 39259854

[ref14] Kim J. Yoo G. Lee T. Kim J. H. Seo D. M. Kim J. (2022). Classification model for diabetic foot, necrotizing fasciitis, and osteomyelitis. Biology 11:1310. doi: 10.3390/biology11091310, PMID: 36138789 PMC9495746

[ref15] Kremers H. M. Nwojo M. E. Ransom J. E. Wood-Wentz C. M. Melton L. J. Huddleston P. M. (2015). Trends in the epidemiology of osteomyelitis: a population-based study, 1969 to 2009. J. Bone Joint Surg. Am. 97, 837–845. doi: 10.2106/jbjs.N.01350, PMID: 25995495 PMC4642868

[ref16] Liu Y. Zhao K. Liu Y. Sun Y. H. Li M. X. Yu M.. (2024). Bone and joint infection complicated with sepsis in neonates and infants under three months of age. J. Pediatr. 100, 156–162. doi: 10.1016/j.jped.2023.09.003, PMID: 37837994 PMC10943287

[ref17] Manz N. Krieg A. H. Buettcher M. Ritz N. Heininger U. (2020). Long-term outcomes of acute osteoarticular infections in children. Front. Pediatr. 8:587740. doi: 10.3389/fped.2020.587740, PMID: 33335875 PMC7737431

[ref18] Manz N. Krieg A. H. Heininger U. Ritz N. (2018). Evaluation of the current use of imaging modalities and pathogen detection in children with acute osteomyelitis and septic arthritis. Eur. J. Pediatr. 177, 1071–1080. doi: 10.1007/s00431-018-3157-3, PMID: 29728840

[ref19] McNeil J. C. (2020). Acute Hematogenous osteomyelitis in children: clinical presentation and management. Infect Drug Resist 13, 4459–4473. doi: 10.2147/idr.S257517, PMID: 33364793 PMC7751737

[ref20] McNeil J. C. Vallejo J. G. Kok E. Y. Sommer L. M. Hultén K. G. Kaplan S. L. (2019). Clinical and microbiologic variables predictive of orthopedic complications following *Staphylococcus aureus* acute hematogenous osteoarticular infections in children. Clin. Infect. Dis. 69, 1955–1961. doi: 10.1093/cid/ciz109, PMID: 30753346 PMC7348234

[ref21] Paliwal A. K. Sahdev R. Deshwal A. Ram B. (2021). Role of ultrasound in the diagnosis of paediatric acute osteomyelitis. J Ultrason 21, e34–e40. doi: 10.15557/JoU.2021.0005, PMID: 33791114 PMC8008204

[ref22] Russell C. D. Ramaesh R. Kalima P. Murray A. Gaston M. S. (2015). Microbiological characteristics of acute osteoarticular infections in children. J. Med. Microbiol. 64, 446–453. doi: 10.1099/jmm.0.000026, PMID: 25596125

[ref23] Schmitt S. K. (2017). Osteomyelitis. Infect. Dis. Clin. N. Am. 31, 325–338. doi: 10.1016/j.idc.2017.01.010 28483044

[ref24] Section J. Gibbons S. D. Barton T. Greenberg D. E. Jo C. H. Copley L. A. (2015). Microbiological culture methods for pediatric musculoskeletal infection: a guideline for optimal use. J. Bone Joint Surg. Am. 97, 441–449. doi: 10.2106/jbjs.N.00477, PMID: 25788299

[ref25] Stephan A. M. Faino A. Caglar D. Klein E. J. (2022). Clinical presentation of acute osteomyelitis in the pediatric emergency department. Pediatr. Emerg. Care 38, e209–e213. doi: 10.1097/pec.0000000000002217, PMID: 32881826

[ref26] Stephan A. M. Platt S. Levine D. A. Qiu Y. Buchhalter L. Lyons T. W.. (2024). A novel risk score to guide the evaluation of acute hematogenous osteomyelitis in children. Pediatrics 153:3153. doi: 10.1542/peds.2023-063153, PMID: 38239108

[ref27] Vaswani A. Shazeer N. Parmar N. Uszkoreit J. Jones L. Gomez A.N.. (2017). Attention is all you need. Advances in neural information processing systems. 30.

[ref28] Walter N. Bärtl S. Alt V. Rupp M. (2021). The epidemiology of osteomyelitis in children. Children 8:1000. doi: 10.3390/children8111000, PMID: 34828711 PMC8621985

[ref29] Wang J. Zhanghuang C. Tan X. Mi T. Liu J. Jin L.. (2022). A nomogram for predicting Cancer-specific survival of osteosarcoma and Ewing's sarcoma in children: a SEER database analysis. Front. Public Health 10:837506. doi: 10.3389/fpubh.2022.837506, PMID: 35178367 PMC8843936

[ref30] Wen Y. Wang C. Jia H. Liu T. Yu J. Zhang M. (2023). Comparison of diagnosis and treatment of MSSA and MRSA osteomyelitis in children: a case-control study of 64 patients. J. Orthop. Surg. Res. 18:197. doi: 10.1186/s13018-023-03670-3, PMID: 36915118 PMC10012508

[ref31] Woods C. R. Bradley J. S. Chatterjee A. Copley L. A. Robinson J. Kronman M. P.. (2021). Clinical practice guideline by the pediatric infectious diseases society and the infectious diseases society of America: 2021 guideline on diagnosis and management of acute hematogenous osteomyelitis in pediatrics. J Pediatric Infect Dis Soc 10, 801–844. doi: 10.1093/jpids/piab027, PMID: 34350458

[ref32] Zaki I. Morrison W. B. (2024). Osteomyelitis and septic arthritis of the foot and ankle: imaging update. Clin. Podiatr. Med. Surg. 41, 745–758. doi: 10.1016/j.cpm.2024.04.007, PMID: 39237182

